# Lack of Galectin-3 Disturbs Mesenteric Lymph Node Homeostasis and B Cell Niches in the Course of *Schistosoma mansoni* Infection

**DOI:** 10.1371/journal.pone.0019216

**Published:** 2011-05-06

**Authors:** Felipe L. Oliveira, Camila Brand, Adelzon A. Paula, Kátia D. Arcanjo, Daniel K. Hsu, Fu-Tong Liu, Christina M. Takiya, Radovan Borojevic, Roger Chammas, Márcia C. El-Cheikh

**Affiliations:** 1 Laboratório de Proliferação e Diferenciação Celular, Instituto de Ciências Biomédicas, Universidade Federal do Rio de Janeiro, Rio de Janeiro, Brazil; 2 Department of Dermatology, School of Medicine, University of California Davis, Sacramento, California, United States of America; 3 Laboratório de Patologia Celular – Instituto de Ciências Biomédicas – Universidade Federal do Rio de Janeiro, Rio de Janeiro, Brazil; 4 Laboratório de Oncologia Experimental, Departamento de Radiologia e Oncologia, Faculdade de Medicina, Universidade de São Paulo, São Paulo, Brazil; 5 Instituto do Câncer do Estado de São Paulo, São Paulo, Brazil; The George Washington University Medical Center, United States of America

## Abstract

Galectin-3 is a β-galactoside-binding protein that has been shown to regulate pathophysiological processes, including cellular activation, differentiation and apoptosis. Recently, we showed that galectin-3 acts as a potent inhibitor of B cell differentiation into plasma cells. Here, we have investigated whether galectin-3 interferes with the lymphoid organization of B cell compartments in mesenteric lymph nodes (MLNs) during chronic schistosomiasis, using WT and galectin-3^-/-^ mice. *Schistosoma mansoni s*ynthesizes GalNAcβ1-4(Fucα1-3)GlcNAc(Lac-DiNAc) structures (N-acetylgalactosamine β1-4 N-acetylglucosamine), which are known to interact with galectin-3 and elicit an intense humoral response. Antigens derived from the eggs and adult worms are continuously drained to MLNs and induce a polyclonal B cell activation. In the present work, we observed that chronically-infected galectin-3^-/-^ mice exhibited a significant reduced amount of macrophages and B lymphocytes followed by drastic histological changes in B lymphocyte and plasma cell niches in the MLNs. The lack of galectin-3 favored an increase in the lymphoid follicle number, but made follicular cells more susceptible to apoptotic stimuli. There were an excessive quantity of apoptotic bodies, higher number of annexin V^+^/PI^-^ cells, and reduced clearance of follicular apoptotic cells in the course of schistosomiasis. Here, we observed that galectin-3 was expressed in non-lymphoid follicular cells and its absence was associated with severe damage to tissue architecture. Thus, we convey new information on the role of galectin-3 in regulation of histological events associated with B lymphocyte and plasma cell niches, apoptosis, phagocytosis and cell cycle properties in the MLNs of mice challenged with *S.mansoni*.

## Introduction

Schistosomiasis is a helminth disease that affects more than 200 million people predominantly in developing countries [1]. *Schistosoma mansoni* infection is a long lasting inflammatory reaction characterized by the presence of adult worms living in the mesenteric venous system, depositing their eggs in small submucosal veins of the intestine. Some of these eggs are washed through the portal blood flow into the liver, where they cause granulomatous inflammatory reactions [2]. A typical Th2 response is well defined in the acute phase and the evolution towards the chronic phase is associated with a down-regulation of several aspects of the immune response to parasites [3]. Egg and worm antigens are continuously drained to mesenteric lymph nodes (MLNs), where they induce an intense polyclonal B cell activation and germinal center reaction in the lymphoid follicles (LFs), concomitant with development of splenomegaly [2], [4], [5].

Lymph nodes have a well-defined lymphoid architecture: a cortical region consisting mostly of B lymphocytes, macrophages and follicular dendritic cells (FDCs) densely packed and organized into LFs; a paracortical region (deep cortex) presenting predominantly T lymphocytes, scarce B lymphocytes and dendritic cells; and a medullary region formed by macrophages and plasma cells organized in cellular cords, besides sinuses that conduct the lymph, cells and secreted immunoglobulin to the venous blood system [6]. This structural organization contributes to B cell activation and proliferation (B220 or CD45RA^+^ cells) into LFs, establishing germinal centers and/or inducing the B cell differentiation into plasmablasts (CD138^+^) and Blimp-1^+^ immunoglobulin secreting plasma cells [7]–[9]. Part of these activated B cells undergo apoptosis and are eliminated by macrophages or resident immature dendritic cells [10]–[12].

During schistosomiasis, both eggs and the adult worms synthesize GalNAcβ1-4(Fucα1-3)GlcNAc(Lac-DiNAc) structures (N-acetylgalactosamine β1-4 N-acetylglucosamine) that interact with galectin-3. The latter is a conserved β-galactoside-binding protein expressed by macrophages that can elicit an intra-hepatic granulomatous reaction and a vigorous humoral immune response [13], [14]. This lectin regulates cell-cell and cell-extracellular matrix interactions, cell signaling, inflammatory responses and biological events, such as cellular activation, migration, differentiation, apoptosis and tumor metastasis [15]. Moreover, galectin-3 acts as a powerful pro-inflammatory molecule to myeloid cells by inducing chemotaxis of monocytes and phagocytosis by macrophages [16], [17]. It also controls T cell activation, proliferation and death [18], [19], modulates carbohydrate-dependent thymocyte interactions in thymic microenvironments [20], and inhibits conventional/B2 and peritoneal/B1 lymphocytes differentiation into plasma cells [21]–[23].

Recently, we showed that one of the hallmarks of *S.mansoni-*infected galectin-3^−/−^ mice is disturbed plasmacytogenesis involving the spleen, bone marrow and MLNs [22]. The increase of plasma cells in the MLNs associated with the continuous arrival of mesenteric antigens could disturb the tissue organization of the lymphoid compartments. Thus, in this work, we investigated the possible interference of galectin-3 in the organization of MLNs in the course of chronic murine schistosomiasis. It was observed that in infected galectin-3^−/−^ mice there was significant histological disorganization in the B and plasma cell niches, which correlated with abnormal cell survival rate and inadequate clearance. We propose that galectin-3 contributes to the maintenance of MLN architecture and drives immune responses by regulating B cell differentiation during *S. mansoni* infection.

## Materials and Methods

### Mice and Schistosoma mansoni infection

Inbred C57/bl6 wild type (WT) and galectin-3^−/−^ mice (backcrossed to C57/bl6 for 10 generations) [24] matched by age and sex were obtained from a colony bred at the Federal University of Rio de Janeiro (Brazil). All mice procedures were performed in accordance with institutional guidelines (protocol number DAHEICB 009, Federal University of Rio de Janeiro). Uninfected mice were used as controls. Thirty day-old mice were infected by percutaneous penetration of 40 *S. mansoni* cercariae (BH strain, Oswaldo Cruz Institute, Rio de Janeiro, Brazil). Mice were euthanized using a carbon dioxide chamber 90–95 days after infection, corresponding to the chronic phase of the disease [25].

### Cell suspensions and flow cytometry

Cell suspensions from MLNs of infected WT and galectin-3^−/−^ mice were obtained *ex vivo* by standard mechanical procedures and washed twice with Phosphate Buffer Solution (PBS), pH 7.2, containing 3% Fetal Bovine Serum (FBS), quantified and their concentration adjusted to 1×10^6^ cells/mL for flow cytometry analysis. The cells were incubated with Fc blocker (Clone 2.4G2) for 10 min before adding the following monoclonal antibodies: FITC anti-B220, anti-Mac 1 and anti-CD4; PE anti-CD19, anti-CD8 and anti-CD-138; PE Cy5.5 anti-Mac1, anti-Gr-1 and anti-B220 (BD Bioscience, USA). The samples were assayed in a flow cytometer (FACSCalibur, BD Bioscience, USA) and the resulting data analyzed using the CellQuest and WinMDI 2.9 software packages. DNA-content was measured by propidium iodide labeling using Vindelov solution [26].

### Phenotype of lymph nodal cells

Lymph node cells were characterized according to phenotypic markers, as follows: monocytes (CD19^−^ B220^−^ Mac-1^+^ Gr-1^+/low^ CD4^−^ CD8^−^ CD138^−^), macrophages (CD19^−^ B220^−^ Mac-1^+^ Gr-1^−^ CD4^−^ CD8^−^ CD138^−^), granulocytes (CD19^−^ B220^−^ Mac-1^+^ Gr-1^+/high^ CD4^−^ CD8^−^ CD138^−^), CD4^+^ T cells (CD19^−^ B220^−^ Mac-1^−^ Gr-1^−^ CD4^+^ CD8^−^ CD138^−^), CD8^+^ T cells (CD19^−^ B220^−^ Mac-1^−^ Gr-1^−^ CD4^−^ CD8^+^ CD138^−^), plasmacytoid dendritic cells (CD19^−^ B220^+^ Mac-1^−^ Gr-1^+^ CD4^+^ CD8^−^ CD138^−^), B2/conventional B cells (CD19^+^ B220^+/high^ Mac-1^−^ Gr-1^−^ CD4^−^ CD8^−^ CD138^−^), B1 cells (CD19^+^ B220^+/low^ Mac-1^+^ Gr-1^−^ CD4^−^ CD8^−^ CD138^−^) and plasma cells (CD19^−^ B220^−/low^ Mac-1^−^ Gr-1^−^ CD4^−^ CD8^−^ CD138^+^).

### Histological preparations

For histological analyses, WT and galectin-3^−/−^ mice were sacrificed during the chronic phase of schistosomiasis infection (5 animals per group). Mesenteric lymph nodes were removed, cut into 0.5 mm-thick slices, washed in cold saline and fixed in 10% buffered formalin fixative. After 12 h of fixation, specimens were dehydrated in alcohol and embedded in paraffin. Sections of 5 µm were obtained and stained with hematoxylin & eosin (H&E). Uninfected WT and galectin-3^−/−^ mice were used as control groups.

### Quantification of lymphoid follicles

LFs were characterized as well-defined rounded clusters containing lymphocyte-like cells and they were quantified per microscopic field using the Image J software (original magnification, 25X). For each experiment, sectioned samples were obtained from mesenteric lymph nodes of five WT and galectin-3^−/−^ mice, both uninfected and infected with *S. mansoni*.

### Immunohistochemistry

Paraffin-embedded sections were de-waxed and hydrated. After inhibition of endogenous peroxidase, sections were incubated for 1 h with 0.01 M PBS containing 5% BSA, 4% skim milk, 0.1% Triton X-100 (Sigma Aldrich, USA), 0.05% Tween-20, and 10% normal goat serum and incubation with the following purified antibodies: anti-gal-3 (clone M3/38; American Type Culture Collection, Manassas, VA, USA, at 1∶10 in PBS, 3% BSA and 1% normal goat serum), anti-B220, anti-CD138 and Blimp-1 (Santa Cruz Biotechnology, USA) overnight at 4°C in a humid chamber. Antibodies were detected with a biotinylated anti-rat IgG (BA-4001, Vector Laboratories, Burlingame, CA, USA) and developed with avidin-peroxidase (1∶50 in PBS) (Sigma Aldrich, USA), using diaminobenzidyne as the chromogen. Sections were counterstained with Harris' hematoxylin. Bright-field pictures were acquired using an Evolution MP 5.0 RTV Color camera (Media Cybernetics, Canada). As negative controls, sections of WT and knockout mice tissue were incubated with non-immune rat serum instead of anti-galectin-3 antibody.

### Apoptosis and Phagocytosis assays

MLNs from WT and galectin-3^−/−^ mice were dissociated and the cells were cultured in RPMI 1640 medium supplemented with 10% SFB in 12-well plates (Corning, USA) for 2 h at 37°C and 5% CO_2_ atmosphere. The non-adherent cells were collected and induced to apoptosis by heating at 43°C for 60 minutes [27]. Subsequently, the apoptotic and dead cells were marked with annexin V-FITC and propidium iodide (PI), and quantified by flow cytometry. Adherent cells were obtained and maintained at 37°C. Apoptotic-induced non-adherent cells were co-cultured with these adherent cells (ratio of 4 non-adherent to 1 adherent cells) during 24 h and 72 h days, at 37°C and 5% CO_2_ atmosphere. The floating cells were washed out and the resting cells were stained by the May-Grunwald-Giemsa method [28]. Adherent-phagocytic cells were identified by the formation of translucent vacuoles and phagosomes inside the cytoplasm and differentiated from the adherent non-phagocytic cells by the absence of them. Results represent a mean of three independent experiments performed using MLNs from infected WT and galectin-3^−/−^ mice. Images were captured using a QColor-3 camera (Olympus, Japan) and analyzed with the Q-Capture software.

### Immunofluorescence to MOMA-2 marker

Direct immunofluorescence staining of MLNs was carried out after de-waxing and rehydration of sections. Auto-fluorescence and charge affinity were inhibited by 0.06% potassium permanganate and 50 mM ammonium chloride. Triton 0.3% - BSA 5% was used to block possible non-specific binding before incubation with the Alexa 488-conjugated anti-MOMA-2 monoclonal antibody (Serotec, USA) overnight at 4°C in a humid chamber. Sections were counterstained with DAPI and visualized using an Olympus IX81 confocal microscope (Olympus, Japan). Images were acquired using the Cell M software (Olympus, Japan).

### Statistical Analysis

The statistical tests were accomplished using the Tukey's multiple comparison test (*t*-test); significance threshold was fixed at *p*≤0.05.

## Results

**Figure 1:**
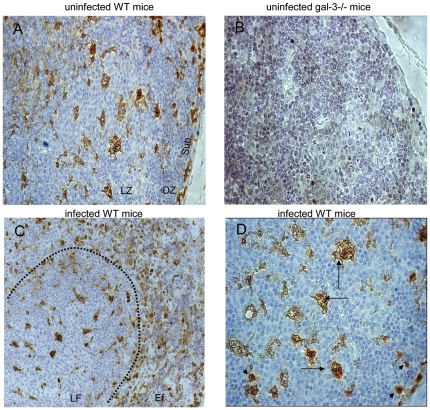
Immunohistochemistry to galectin-3 in MLNs. (A) The immunoreactivity for galectin-3 was preferentially found in follicular non-lymphoid cells from uninfected wild-type mice. (B) Galectin-3 was not detected in MLNs of uninfected galectin-3−/− mice. (C) Photomicrograph of lymphoid follicle of MLNs surrounded by a dotted line derived from infected wild-type mice. (D) Galectin-3+ cells have distinct morphology and intensity of immunoreaction. The arrows indicate elongated and vacuolated cells, while the arrowheads point to smaller and rounded cells. Original magnification: A and C, 200x; B and D, 400x. Data are representative of three independent experiments. LZ: light zone; DZ: dark zone; Sub: subcapsular region.

Galectin-3 has been reported as a modulatory molecule that regulates B cell differentiation into plasma cells [21]–[23]. First, we evaluated galectin-3^+^ cells in the MLNs of uninfected and infected mice in the chronic phase of *S.mansoni-*infection. In uninfected WT mice, we observed elongated and vacuolated galectin-3^+^ cells predominantly in subcapsular sites and in the light zone of the LFs (Figure 1A). As expected, galectin-3^+^ cells were not detected in samples obtained from galectin-3 deficient mice (Figure 1B). In infected WT mice, we also observed large follicular galectin-3^+^ cells (Figure 1C, LF region) and a significant number of small and rounded galectin-3^+^ cells in extrafollicular regions (Figure 1C, Ef region). In more details, we observed that the majority of these galectin-3^+^ cells within the LFs were non-lymphoid cells (Figure 1D). These data indicate that galectin-3 was expressed by follicular and extrafollicular cells in the MLNs in uninfected conditions and the expression of this lectin seems increased in distinct cell types dispersed by the parenchyma of the MLNs obtained from *S.mansoni*-chronically infected mice.

Previously, we described that total leukocyte number in the MLNs was not modified when comparing WT and galectin-3^−/−^ uninfected mice [22]. However, the cellularity was drastically modified in the MLNs of chronically infected galectin-3^−/−^ mice. In the absence of galectin-3, granulocytes and monocytes were significantly increased during chronic phase of the disease. In contrast, macrophages, TCD8^+^ cells, and B lymphocytes were drastically decreased in these mice, compared with infected WT mice (Table 1).

**Table 1:** Absolute number of the cell subsets in the mesenteric lymph nodes of WT and galectin-3−/− mice infected with *S.mansoni.*

*Cell types*	*WT mice*	*Gal-3−/− mice*
Total cells	86.6×10^6^ cells/mL±11.42	63.2×10^6^ cells/mL±6.35 *
Granulocytes	19.1×10^5^ cells/mL±4.7	25.8×10^5^ cells/mL±7.3
Monocytes	12.8×10^5^ cells/mL±2.9	34.5×10^5^ cells/mL±3.6 *
Macrophages	44.4×10^5^ cells/mL±4.8	19.9×10^5^ cells/mL±2.9 *
CD4+ lymphocytes	201.2×10^5^ cells/mL±18.1	219.6×10^5^ cells/mL±21.2
CD8+ lymphocytes	262.4×10^5^ cells/mL±31.3	167.5×10^5^ cells/mL±27.2 *
Total B lymphocytes	343.7×10^5^ cells/mL±21.2	161.9×10^5^ cells/mL±18.9 *

Data are reported as means ± SEM, They are representative of three independent experiments, Statistical analysis: Tukey's multiple comparison test (*, *P*<0.05).

**Figure 2:**
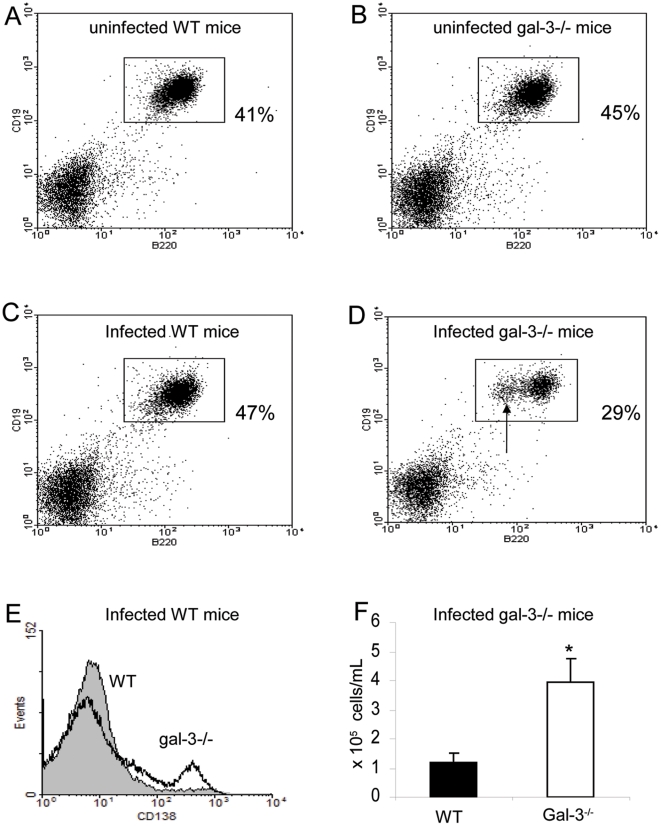
Phenotypic analysis of B lymphocytes in the MLNs. B220+ CD19+ cells were selected and quantified in uninfected wild typeWT and galectin-3−/− mice (A and B, respectively), and in chronically-infected wild typeWT and galectin-3−/− mice (C and D, respectively). (D) The arrow pointed to distinct B220low subpopulation found in the absence of galectin-3. (E) Histograms reflect the surface expression of CD138, a plasma cell marker. Full histogram: WT mice. Empty histogram: galectin-3−/− mice. (F) Absolute number of plasma cells in MLNs of infected WT (solid bars) and infected galectin-3−/− mice (open bars). Data are reported as means + SEM and are representative of three independent experiments, each carried out in five mice with chronic infection. Statistical analysis: Tukey's multiple comparison test (*, P<0.05).

Considering the role of galectin-3 in B cell differentiation [21]–[23], we analyzed the phenotype of B lymphocytes (B220^+^ CD19^+^ B cells) in the MLNs of WT and galectin-3^−/−^ mice. In uninfected mice, we did not find differences in the B lymphocytes (Figure 2A and 2B, respectively). However, there were significant differences in B lymphocyte and plasma cell number in MLNs of chronically-infected mice. In infected galectin-3^−/−^ mice, B lymphocytes were significantly reduced (approximately 29% of the cells), when compared with infected WT mice (about 47% of the cells) in the MLNs (Figure 2C and 2D). Moreover, we clearly found a cell subpopulation expressing lower levels of surface B220 in the absence of galectin-3 (Figure 2D, arrow), suggesting that these cells could be differentiating into B220^−/low^ plasma cells.

To reinforce this proposal, we marked these cells with anti-CD138 and observed that there was an increase of CD138^+^ plasma cells in galectin-3^−/−^ mice when compared with WT mice (Figure 2E). By quantifying the absolute number of plasma cells in the MLNs, we showed that the number of plasma cells in galectin-3−/− mice were four times higher, compared with infected WT mice (Figure 2F). Previously, we demonstrated that the plasma cell number in these lymphoid organs was also similar between these groups of uninfected mice [22]. According with these data, we suggest that the lack of galectin-3 disturbs B lymphocyte-plasma cell homeostasis in the MLNs of the *S.mansoni*-infected mice.

**Figure 3:**
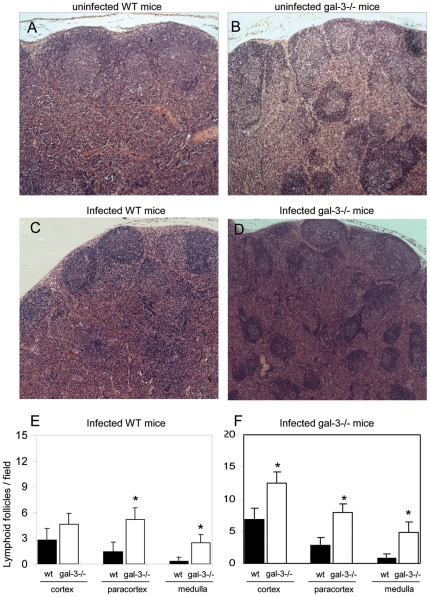
Histological analysis of MLNs of WTwild-type and galectin-3−/− mice. Midsagittal section of MLN showing lymphoid follicles preferentially within the cortex and scarcely in paracortex in uninfected and infected WT mice (A and C, respectively). Histological section from MLNs of uninfected and infected galectin-3−/− mice exhibiting lymphoid follicles randomly scattered throughout the cortex, paracortex and medulla (B and D, respectively). The samples were stained with hematoxilin and eosin. Lymphoid follicles were quantified by microscopic field in uninfected (E) and infected mice (F), with magnification of 25x. The solid bars indicate the WT mice and the open bars represent galectin-3−/− mice. Data are reported as means + SEM and are representative of three independent experiments. Statistical analysis: Tukey's multiple comparison test (*, P<0.05). A–D, original magnification: 200x.

In order to investigate the relationship between the absence of galectin-3 and the imbalance in B lymphocyte and plasma cell populations, we analyzed the lymphoid architecture of the MLNs and the distribution of B lymphocytes and plasma cells *in situ*. In uninfected WT mice, the LFs were normally found in the cortical region (Figure 3A), whereas in galectin-3^−/−^ uninfected mice, the LFs were abnormally dispersed throughout the paracortical and medullary regions (Figure 3B). These follicles were quantified according their location and they are increased in the paracortical and medullary regions in galectin-3^−/−^ mice (Figure 3E). In *S.mansoni*-infected WT mice, the LFs were detected predominantly in the cortical and paracortical sites, being rare or even absent in the medullary region (Figure 3C). MLNs of infected galectin-3^−/−^ mice were widely disorganized, containing LFs scattered all over the cortex, paracortex and medulla, disrupting the classical architecture of these organs (Figure 3D). These follicles were significantly increased in all of these regions, compared with infected WT mice (Figure 3F).

**Figure 4:**
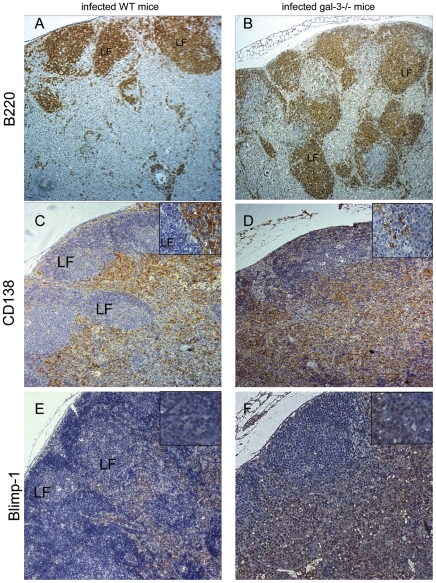
Immunohistochemistry to localize B lymphocyte and plasma cell niches in MLNs. (A) Immunoreactivity for B cells using anti-B220 antibody preferentially within of lymphoid follicles (LF) in chronically-infected WT wild type mice. (B) In galectin-3−/− mice, B220+ cells were randomly dispersed by the parenchyma forming numerous lymphoid follicles. In infected WT mice, CD138+ plasma cells and Blimp-1+ antibody-secreting cells were found in cellular cords in extrafollicular regions (C and E, respectively). In infected galectin-3−/− mice, CD138+ and Blimp-1+ plasma cells were randomly scattered throughout the parenchyma (D and F, respectively). A–F: Original magnification, 200x. Boxed images: original magnification, 400x. Data are reported as means + SEM and are representative of three independent experiments.

Activated B220^+^ B lymphocytes proliferate in the germinal center reaction and a proportion of these cells differentiate into plasma cells (CD138^+^ cells) in extrafollicular sites. Subsequently, plasma cells expressing Blimp-1 differentiate into immunoglobulin secreting cells (Blimp-1^+^ cells) in the medullary region, more precisely, in medullary cellular cords [29], [30]. The atypical tissue organization and the exacerbated plasmacytogenesis observed in MLNs of *Schistosoma*-infected galectin-3^−/−^ mice led us to investigate the micro-anatomical location of B lymphocytes and plasma cells, with an emphasis on the antibody-secreting plasma cells. In contrast to infected WT mice, where most B220^+^ B cells were properly distributed within LFs, B220^+^ B lymphocytes were widely dispersed throughout both intra and extrafollicular regions in the MLNs of galectin-3^−/−^ mice (Figure 4A–4B, respectively). In WT mice, CD138^+^ cells were enriched in well-defined extrafollicular niches forming intense cellular cords (Figure 4C) and Blimp-1^+^ cells were widely scattered throughout paracortical and medullary parenchyma (Figure 4E). In infected galectin-3^−/−^ mice, CD138^+^ and Blimp-1^+^ plasma cells were randomly distributed throughout intra and extra-follicular sites (Figure 4D and 4F, respectively). As the number of B lymphocytes and plasma cells were similar in uninfected WT and galectin-3 mice, we evaluated these niches only in infected mice. Together, these data indicate that the lack of galectin-3 disturbs severely B lymphocyte and plasma cell niches during chronic phase of schistosomiasis.

**Figure 5:**
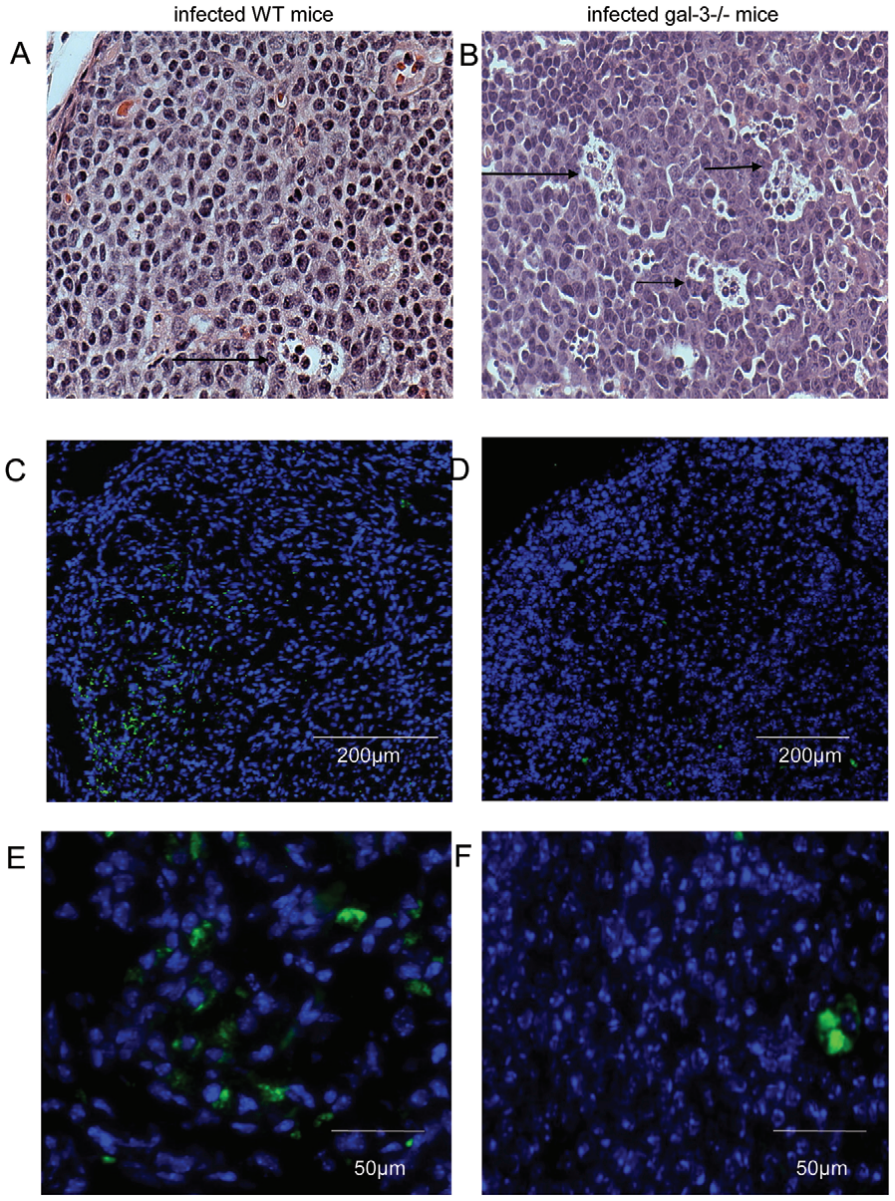
Histological analysis of lymphoid follicles of MLNs of infected mice. (A) In wild type (WT) mice, section of lymphoid follicles showed scarce apoptotic bodies (arrow). In infected gal-3−/− mice (B), there was high number of cellular debris dispersed throughout the follicles (arrows). Immunofluorescence to MOMA-2+ macrophages. (C) Immunoreactivity for MOMA-2 Alexa 488 (green cells) in MLNs of WT and (D) in galectin-3−/− mice. (E) Detailed MOMA-2+ cell clusters in WT mice and (F) rare MOMA-2+ cells in the absence of galectin-3. The nuclei were stained with DAPI. Data are representative of three independent experiments, each carried out in three mice with chronic infection.

By analyzing the follicular structures closely, we observed that there were higher numbers of apoptotic bodies and cellular debris in LFs of chronically-infected galectin-3^−/−^ mice, when compared with infected WT mice (Figure 5A–5B, arrows). Considering the reduced macrophage number in MLNs of infected galectin-3^−/−^ mice (Table 1), we decided to investigate their distribution using a follicular-specific macrophage-marker. MOMA-2^+^ macrophages were widely distributed throughout the parenchyma of the MLNs of WT mice (Figure 5C and 5E). In contrast, in MLNs of galectin-3^−/−^ mice infected with *S. mansoni*, the quantity of MOMA-2^+^ cells was drastically reduced (Figure 5D and 5F). As these MOMA-2^+^ macrophages are potent phagocytes, we suggest that the lack of galectin-3 is associated with the high number of cellular bodies presented within the LFs.

**Figure 6:**
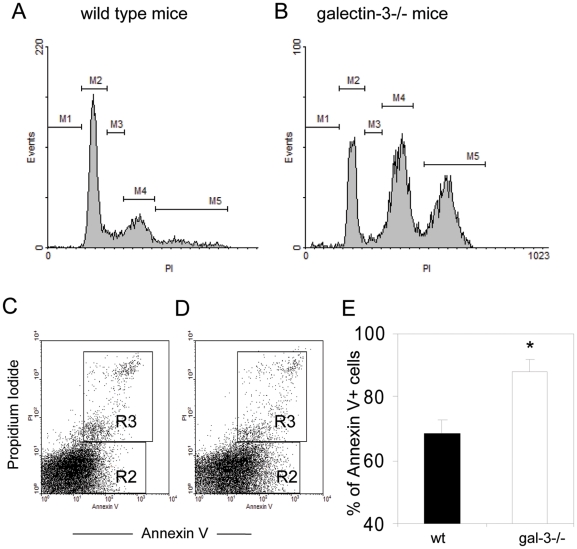
Cell cycle analysis and apoptosis index in MLNs of WT and galectin-3^−/−^ infected mice. Histograms represent the stages of cell cycle in WT (A) and Gal-3^−/−^ mice (B) infected with *S.mansoni*. In both graphs, the phases were described as bellow: M1 - sub G1/G0; M2 – G1/G0; M3 – S phase; M4 – G2/M and M5 – hyperploid cells. (C–D) Quantification of Annexin-V^+^/Propidium iodide (PI) ^−^ apoptotic cells (gated in R2 region) and Annexin-V^+^/PI^+^ dead cells (gated in R3 region), in WT (C) and Gal-3^−/−^ mice (D). (E) Quantification of apoptotic cells induced by high temperature. Solid bars represent WT mice and open bars indicate Gal-3^−/−^ mice. Data are reported as means ± SEM and are representative of three independent experiments. Statistical analysis: Tukey's multiple comparison test (*, *P*<0.05). A–B, original magnification, 400x.

Two major points were still unclear: the reduced cellularity compared with the high quantity of LFs and the raise of cellular bodies in the MLNs of chronically-infected galectin-3^−/−^ mice. Then, we performed the DNA content analysis and annexin-V staining by flow cytometry in lymph nodal cells, since data obtained by these methods could clarify, at least in part, these phenomena. Although we did not observe significant differences between the number of cells in sub-G1/G0 phases on MLNs from WT and galectin-3^−/−^ mice (Figure 6A and 6B, in M1), the effects of the lack of galectin-3 were evident in the other phases of the cell cycle. In WT mice, about 57.7% of the cells were cycling in G1/G0 stages (Figure 6A, in M2) and approximately 22.7% of the cells were in the same phase of the cell cycle in galectin-3^−/−^ mice (Figure 6B, in M2). In parallel, the number of cells in the S phase was significantly increased in MLNs of galectin-3^−/−^ mice (Figure 6A and 6B, in M3). Moreover, in WT mice, approximately 24.8% of the lymph nodal cells were in G2/M phase. In contrast, in infected galectin-3^−/−^ mice, the proportion of the cells was 40.1% (Figure 6A and 6B, in M4). These results suggest that the significant proportion of the cells in MLNs of infected galectin-3^−/−^ mice are arrested in G2 phase of the cell cycle. Besides, we found that 30.8% of the cells obtained from MLNs of galectin-3^−/−^ mice presented an abnormal DNA content, suggesting hyperploidy or the presence of a large amount of aggregates of nuclei derived from dead cells (Figure 6A and 6B, in M5). These values are presented in table 2.

**Table 2:** Relative number of cells during cell cycle events in MLNs of mice chronically-infected with *Schistosoma mansoni.*

*Region*	*WT mice*	*Gal-3−/− mice*	*Cell cycle stage*
M1	0.27%	0.60%	Fragmented DNA
M2	57.71%	22.72%	G1/G0 phases *
M3	9.03%	3.00%	S phase
M4	24.79%	40.13%	G2/M phases *
M5	9.94%	30.85%	Hyperploid *

Data are reported as means ± SEM, They are representative of three independent experiments, Statistical analysis: Tukey's multiple comparison test (*, *P*<0.05).

Although it was clear that the absence of galectin-3 was arresting the cell cycle in G2 phase, it was still undefined if the cellular debris observed was consequence of this disturbed cell cycle. Thus, we used annexin-V and propidium iodide staining to quantify possible apoptotic and dead cells and compared samples of WT and galectin-3^−/−^ mice, both chronically-infected with *S.mansoni*. We observed that approximately 19.9% of the cells were annexin V^+^/PI^neg^ in the MLNs of WT mice (Figure 6C, in R2), whereas about 30.9% of the cells were annexin V^+^/PI^neg^ in the MLNs of galectin-3^−/−^ mice (Figure 6D, in R2). These data suggest that the lack of galectin-3 promotes a significant increase in the number of cells undergoing apoptosis. We did not find differences in the number of annexin V^+^/PI^+^ dead cells when compared WT (5.5% of the cells) and galectin-3^−/−^ mice (4.9% of the cells) (Figure 6C and 6D, in R3). Perhaps, galectin-3 has an anti-apoptotic role in lymph node cells in the course of chronic phase of schistosomiasis.

In order to investigate the possible anti-apoptotic role of galectin-3, we provided apoptotic stimuli by means of raising the temperature in MLNs cells from infected WT and galectin-3^−/−^ mice. The cells of MLNs of both WT and galectin-3^−/−^ mice were induced to apoptosis maintained in a culture system at 43°C during 1 hour. After this time, these cells were stained with annexin-V and propidium iodide (PI). We observed that cells from MLNs of infected galectin-3^−/−^ mice were more susceptible to apoptosis, when compared to their WT counterpart (Figure 6E). Taken together, these data indicate that the lack of galectin-3 disturbs the cell cycle and increased the susceptibility to apoptotic signals on lymph node cells derived from chronically-infected mice. Although these data are all well suggestive, the origin of the cellular debris (significantly increased in infected galectin-3^−/−^ mice) remains unclear.

**Figure 7:**
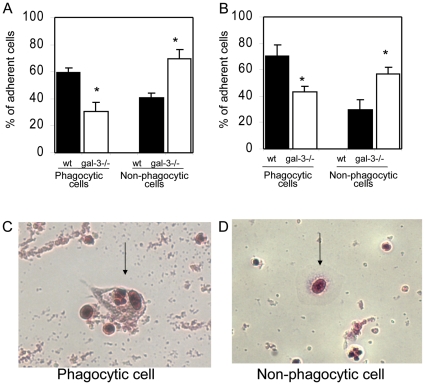
Quantification of phagocytic and non-phagocytic cells of MLNs of WT and galectin-3^−/−^ infected mice. Non-adherent lymph nodal cells were collected, induced to apoptosis by high temperature and co-cultured with adherent cells to be engulffed. Measurement of phagocytic and non-phagocytic cells after 24 h (A) and 72 h (B) of co-culture procedures. The solid bars indicate the wild-type mice and the open bars represent the gal-3^−/−^ mice. Phagocytic cells had a translucent vacuole and phagosomes (C, arrow), while non-phagocytic cells were identified by the absence of phagossomes and clear citoplasm (D, arrow). Data are reported as means ± SEM and are representative of three independent experiments using cells derived from chronically-infected mice. Statistical analysis: Tukey's multiple comparison test (*, *P*<0.05).

Another possibility concerns the disturbed clearance of these cellular debris. To investigate this, we evaluated the phagocytic capacity of phagocytes derived from MLNs of both WT or galectin-3^−/−^ mice. Total lymph node cells were maintained in the culture system, as described in “methodology section”. Non-adherent cells were isolated and treated to induce apoptosis. Adherent cells were maintained in normal conditions of the culture. Subsequently, both non-adherent and adherent cells obtained from either WT or galectin-3^−/−^ mice were co-cultured. After 24 and 72 hr, adherent cells were macrophage-like cells containing central or peripheral nuclei, spread but not elongated morphology and high adherence capacity. The number of actually phagocytic cells was determined according to the presence of intracellular vacuoles containing cellular material or apoptotic bodies. We found a reduced number of phagocytic cells derived from MLNs of infected galectin-3^−/−^ mice, when compared to adherent phagocytic cells obtained from MLNs of infected WT mice. Conversely, non-phagocytic adherent cells without any intracellular apoptotic cells of infected galectin-3^−/−^ mice were predominant, when compared to non-phagocytic adherent cells from WT group (Figure 7A–7B). Representative photomicrographs depict the morphology of phagocytic and non-phagocytic cells are shown in Figure 7C and Figure 7D (arrows). These findings suggest that, besides the increased sensitivity of galectin-3^−/−^ cells to cell death, the higher amount of cellular debris in MLNs from infected galectin-3^−/−^ mice could be a result of an impaired phagocytic capacity in these lymphoid compartments. All these results indicate that the lack of galectin-3 disturbs MLNs homeostasis, leading to disruption of the architecture of B cell compartments during chronic phase of murine schistosomiasis.

## Discussion

Galectin-3 null mice are viable under normal conditions [24], [31] and long lasting inflammatory responses, like Chagas' Disease and Schistosomiasis [21], [22]. *S. mansoni*-infected galectin-3^−/−^ mice display no differences in the parasite burden, egg deposition, parasite survival or fecundity when compared to infected WT mice. However, these knockout mice have an abnormal number of splenic T and B lymphocytes, accelerated plasmacytogenesis and hyperimmunoglobulinemia with high levels of serum IgG and IgE, eosinophilia and distinct intra-hepatic fibrogranulomatous reaction [22], [32].

Galectin-3 is highly expressed by human monocyte differentiating into macrophages [33] and is lowly expressed by human monocytes that differentiate into dendritic cells [34]. In the MLNs homeostasis, the role of galectin-3 it is not clear. In this context, Hoyer and colleagues described that human tonsilar follicular dendritic cells are galectin-3^+^ and these cells regulate anti-apoptotic mechanisms during diffuse large B-cell lymphoma progression [35]. Consistent with that, in murine chronic schistosomiasis model, we observed large and spread galectin-3^+^ follicular cells scattered throughout LFs of MLNs from infected WT mice, while the bulk of rounded lymphocyte-like cells were galectin-3-negative.

MLNs continuously draining the major part of tissues involved by schistosomiasis. In the course of the chronic phase, there is progressive hyperplasia and the lymphoid organization is maintained [25]. In this work, we showed that the basic structural aspects of the MLNs of galectin-3^−/−^ mice were softly disturbed independently of the infection. However, the course of the chronic schistosomiasis significantly amplified these histological disorders and the MLNs of *S. mansoni*-infected galectin-3^−/−^ mice contained macrophage and B2 lymphocyte niches severely modified. Our results are not sufficient to prove whether galectin-3 controls these microenvironments, although it has been described that resident macrophages are responsible for phagocytosis of apoptotic cells [17] and constitutively these cells control the distinct steps of trafficking and differentiation of these B cells [36]. Since *S.mansoni* chronically-infected galectin-3^−/−^ mice had delayed monocyte-macrophage differentiation [22], we propose that the disorganization on B lymphocyte and plasma cell niches is narrowly associated with this eminent macrophage dysfunction. In infected WT mice, B lymphocytes and plasma cells are normally distributed throughout follicular and extrafollicular sites, respectively. However, in infected galectin-3^−/−^ mice, these organizational scenarios are widely modified, where B220^+^ B cells, CD138^+^ plasma cells and Blimp-1^+^ antibody-secreting cells are abnormally misplaced throughout the cortex, paracortex and medullar regions.

It is known that strict mechanisms regulate B cell decision between follicular and extrafollicular areas, where B lymphocytes rapidly differentiate into antibody-secreting cells [37]. Although some light has been shed on this subject, it remains unclear how galectin-3 regulates B cell differentiation into plasma cells. In this context, it was shown that galectin-3 inhibits Blimp-1 expression in different experimental models, interfering with terminal differentiation of B lymphocytes in antibody-secreting plasma cells [21], [23]. The increase of Blimp-1^+^ cells in the absence of galectin-3 endorses the hypothesis that galectin-3 is a potential regulator of Blimp-1 expression.

The macrophage dysfunction can also be associated with the higher rate of cell death and reduced phagocytosis levels due to the absence of galectin-3, where we did observe a significant histological disorder in the distribution of MOMA-2^+^ macrophages. These cells were described as typical tissue macrophages predominantly detected in subcapsular sinus, follicles (tingible body macrophages) and throughout paracortical and medullary regions [38]. By definition, tingible body macrophages are large phagocytic cells containing many apoptotic cells in distinct states of degradation [39]. In this work, we demonstrated that the number of total and MOMA-2^+^ macrophages are both decreased and these macrophages have reduced phagocytic capacity to engulf apoptotic cells in the MLNs from infected galectin-3^−/−^ mice.

In accordance, Miyake and colleagues showed that injected dead cell bodies were rapidly engulfed by macrophages in the splenic marginal zone, indicating a critical role of macrophages in quickly removing apoptotic residues [40]. During schistosomiasis, soluble eggs antigens (SEA) favor the activation-induced cell death of follicular B and T lymphocytes [41]. Here, we did find an elevated number of cellular debris inside of LFs in the MLNs of chronically-infected galectin-3^−/−^ mice. Thus, we propose that, at least in part, galectin-3 plays a regulatory role in anti-apoptotic events and/or phagocytosis of dead cells during schistosomiasis.

DNA content analysis findings corroborate the cellularity results. We found that the total cell number was significantly reduced in the MLNs of infected galectin-3^−/−^ mice. Analyzing the cell cycle data, we did note that cells arrested in G1 stage were numerically reduced, while in G2 stage, the cellularity was increased. Possibly, the absence of galectin-3 downregulates mitotic cycles and favors the generation of cells more susceptible to apoptosis. Paradoxically, LFs in the MLNs of infected galectin-3^−/−^ mice were more numerous, but the majority presented suggestive lower cellular density and apoptotic bodies accumulated inside them.

In conclusion, we provide clues on the role of galectin-3 in driving histological changes in MLNs of mice infected with *S.mansoni*. We suggest that the tissue disorganization observed in the absence of galectin-3 is, at least partially, responsible for an abnormal immune regulation and changes in cell number and activities, including survival, apoptosis, phagocytosis, and differentiation. Nevertheless, tissue damages and/or lost of appropriate cell interactions and constrains in lymphoid tissue might contribute to some aspects of immune response against to helminths, as well as tumorigenesis and progression of lymphoproliferative diseases, such as leukemia and lymphomas.
